# Heterogeneity of Molecular Characteristics among *Staphylococcus argenteus* Clinical Isolates (ST2250, ST2793, ST1223, and ST2198) in Northern Taiwan

**DOI:** 10.3390/microorganisms8081157

**Published:** 2020-07-30

**Authors:** Jia-Chuan Hsu, Tsai-Wen Wan, Hao Lee, Xiao-Mei Wang, Yu-Tzu Lin, Chiau-Jing Jung, Tai-Fen Lee, Po-Ren Hsueh, Lee-Jene Teng

**Affiliations:** 1 Department of Clinical Laboratory Sciences and Medical Biotechnology, National Taiwan University College of Medicine, Taipei 100, Taiwan; d03424005@ntu.edu.tw (J.-C.H.); candy09430926@hotmail.com (T.-W.W.); b97404042@ntu.edu.tw (H.L.); hsmei508@gmail.com (X.-M.W.); fen030175@gmail.com (T.-F.L.); 2 Department of Laboratory Medicine, National Taiwan University Hospital, National Taiwan University College of Medicine, Taipei 100, Taiwan; hsporen@ntu.edu.tw; 3 Department of Medical Laboratory Science and Biotechnology, China Medical University, Taichung 404, Taiwan; yutzulin@mail.cmu.edu.tw; 4 Department of Microbiology and Immunology, School of Medicine, College of Medicine, Taipei Medical University, Taipei 110, Taiwan; cjjung@tmu.edu.tw; 5 Department of Internal Medicine National Taiwan University Hospital, National Taiwan University College of Medicine, Taipei 100, Taiwan

**Keywords:** *Staphylococcus argenteus*, *Staphylococcus aureus*, ST2250, ST2793, ST1223, ST2198, CRISPR

## Abstract

*Staphylococcus argenteus* is an emerging pathogen that is recognized as non-pigmented *Staphylococcus aureus*. However, the molecular characteristics of *S. argenteus* and its virulence factors have not been well studied. The present study analyzed 96 isolates of *S. argenteus* recovered from blood. Identification of *S. argenteus* was based on results of MALDI-TOF MS and lacking *crtM* gene. All 96 isolates were methicillin-susceptible. Multilocus sequence typing (MLST) revealed four sequence types: ST2250 (*n* = 72), ST2793 (*n* = 12), ST1223 (*n* = 10), and ST2198 (*n* = 2). All 72 ST2250 isolates harbored CRISPR loci with polymorphism of direct repeats and spacers, but no other STs carried CRISPR loci. To date, ST2793 isolates have rarely been reported in other countries. Collagen-binding adhesin gene (*cna*) and staphylococcal enterotoxin type C (*sec*) were detected in 12 (100%) and 8 (67%) ST2793 isolates, respectively. ST1223 has been reported as food poisoning pathogens, and enterotoxin gene clusters (*egc*) were detected in all 10 isolates, while *seb* gene was detected in three isolates. Two ST2198 isolates carried bone sialoprotein-binding protein gene (*bbp*), belonging to *agr* type IV. Our focus on the heterogeneity of molecular characterization in four ST types of *S. argenteus* revealed that *S. argenteus* had been isolated as early as 2000. Each ST type of *S. argenteus* harbors particular genetic markers that may contribute to their virulence.

## 1. Introduction

*Staphylococcus argenteus* is an emerging pathogen that has been recognized as non-pigmented *Staphylococcus aureus* displaying white colonies on chocolate agar plates owing to lack of the *crtOPQMN* gene operon required for staphyloxanthin pigment production. [1,2]. To date, *S. argenteus* clinical isolates have been reported in many countries, including Belgium, France, Thailand, Japan, China, and Taiwan [3,4,5,6,7,8]. However, the molecular characterization and the virulence factors of *S. argenteus* have not been well studied.

*S. argenteus* was proposed in 2015, and it is a divergent lineage branching from *S. aureus,* with approximately 10% nucleotides divergence or 87% average nucleotides identity (ANI) [2]. *S. argenteus* is a Gram-positive, catalase-positive, and coagulase positive cocci and demonstrates β-hemolysis on blood agar [2]. The type strain of *S. argenteus* MSHR1132^T^ (= DSM 28299^T^) was isolated from the blood culture of an indigenous patient in 2006 in Darwin, Northern Territory, Australia, and it belongs to ST1850, methicillin-resistant and initially recognized as clonal complex 75 (CC75) [2]. Phenotypic identification of *S. argenteus* is difficult since most biochemical phenotypes of *S. argenteus* are similar to those of *S. aureus,* including coagulase activity.

To date, characteristics such as the lack of pigment have been applied to identify *S. argenteus* along with multilocus sequence typing (MLST), *rpoB*, nonribosomal peptide synthetase (*NRPS*) gene molecular typing, and/or MALDI-TOF MS [5,7,9]. Recently, molecular characterization of whole genome sequencing or genotyping of *S. argenteus* with more than ten clinical isolates of *S. argenteus* have been reported in Denmark, Thailand, China, Sweden, and Japan [10,11,12,13,14]. Previous studies report the prevalence rate of *S. argenteus* as ranging from <1% in European countries and Japan to 19% of community onset Staphylococcus sepsis in Thailand [10,14,15]. ST2250 lineage is the predominant clone and is found worldwide [10,11,12,13,14]. The second most prevalent is ST1223, and its prevalence rate is apparently lower than ST2250 [14]. However, ST2793 or ST2198 are found only sporadically in Europe and Asia [10,14]. In addition, *S. argenteus* infections in humans may be linked mainly to community-onset with high mortality [8,15].

Previous studies have indicated that *S. argenteus* is generally more susceptible to antibiotics compared to *S. aureus.* [15] Some *S. argenteus* isolates may harbor virulence factors such as Panton-Valentine leucocidin (PVL) or enterotoxin (-like) genes [4,10,14,15,16,17]. *S. argenteus* has been isolated in Taiwan as reported by Chen et al. [8,9] and Chu et al. [18]. The most prevalent lineage is that of ST2250 [9], which is similar to that shown in other counties. However, it is unclear whether each ST of *S. argenteus* may display different characterization and may delineate its clinical significance and pathogenicity.

The purpose of this study was to examine molecular characteristics of 96 *S. argenteus* comprising four STs isolated from northern Taiwan. The results indicated that each ST type of *S. argenteus* harbors particular genetic markers that may contribute to their virulence.

## 2. Materials and Methods

### 2.1. Bacterial Isolates and Identification of S. argenteus by Detection of MALDI-TOF MS, MLST Typing, and Detection of CrtM

Bacterial isolates were collected from the National Taiwan University Hospital (NTUH) in 2000, 2005, and 2010–2012 in prior studies [8,9]. In brief, NTUH is a 2500-bed teaching hospital providing both primary and tertiary care in northern Taiwan. For all the preserved *S. argenteus* (previously misidentified as *S. aureus*) isolates used in this study, species identification was performed by colony morphology, Gram staining results, a positive slide or tube coagulase test, and using the Vitek 2 identification system (bioMerieux, Marcy l’Etoile, France). Antimicrobial susceptibility was determined using the standard disk diffusion method and Vitek 2 identification system. In total, 96 methicillin-susceptible *S. argenteus* (MSSAg) bloodstream isolates were further confirmed by MALDI-TOF MS, multilocus sequence typing (MLST), and detection of the *crtM* gene. MLST was conducted as previously described [19], and STs were assigned using the *S. aureus* MLST database (http://saureus.mlst.net). The previously described amplification PCR primers were crtMupF (5′-TTAGGAAGTGCATATACTTCAC-3′) and crtMdownR (5′-GGCACCGTTATACGATCATCGT-3′), and conditions were established to amplify a partial *crtM* gene involved in staphyloxanthin production [20]. The 1660-bp amplicon was generated in isolates containing *crtM*. The existence of the *crt* operon was detected by PCR with primers crtOp-F (5′-CCATGAAAAGCACCATATTT-3′) and crtOp-R (5′-GTTAACAGCAACGGTTCTGT-3′), whose targets on the *crt* operon are upstream and downstream, respectively. Amplification was performed using the following conditions: 7 min at 94 °C, followed by 30 cycles at 94 °C for 30 s, 45 °C for 30 s, and 68 °C for 6 min, and ending with a final extension time of 10 min at 68 °C. A 6.4 kb amplicon was obtained in pigmented isolates, while a 977 bp amplicon was generated from non-pigmented isolates. For matrix-assisted laser desorption/ionization time-of-flight mass spectrometer (MALDI-TOF MS, Bruker Daltonik GmbH, Bremen, Germany) identification, all *S. argenteus* isolates were prepared and analyzed as previously described [9].

### 2.2. Pulsed-Field Gel Electrophoresis and Spa Typing

The genetic associations of 96 *S. argenteus* isolates were determined by pulsed-field gel electrophoresis (PFGE). The DNA in gel plugs was digested with SmaI (New England BioLabs, Ipswich, MA, USA) and then separated in a CHEF-DR III apparatus (Bio-Rad Laboratories, Inc., Hercules, CA, USA). The plugs were applied to wells of 0.8% (*w*/*v*) agarose gels (Bio-Rad Laboratories, Inc., Hercules, CA, USA). PFGE was carried out at 200 V and 12 °C for 20 h, with a pulse angle of 120° and pulse times ranging from 5 to 60 s. The pulsotypes were analyzed using BioNumerics software version 4.0. (Applied Maths, Sint-MartensLatem, Belgium) In addition, the *spa* typing was determined by PCR and sequencing, as previously described [21]. The X-region of the staphylococcal protein A gene (*spa* gene) was amplified with primer pair spa-1095F and spa-1517R [21]. The resulting *spa* types were assigned using the Ridom Spaserver website (http://www.spaserver.ridom.de) [22].

### 2.3. Coa, DnaJ, GroEL Gene and Spacer Sequencing, and AgrD Type

The previously described universal amplification PCR primers SA-(F) (5′-GCCAAAAGAGACTATTATGA-3′), SA-(R) (5′-ATTGYTTACCYGTTTGTGTACC-3′), Gor600F (5′-GGNGAYGGNACNACNACNGCNACNGT-3′), Gor600R (5′-TCNCCRAANCCNGGYGCNTTNACNGC-3′), groESL-F (CACCACGTAACATWGMTTGWC), groESL-R (TCGTSTTCCAACAATWYGCWGG) and conditions were established to amplify the partial *dnaJ* (*hsp40*), *groEL* (*hsp60*), and spacer gene [23,24]. In addition, the amplified product was sequenced. For the selected isolates, the accessory gene regulator (*agr*) group was determined by the PCR with specific primers and sequenced for *agrB* region [25]. Staphylocoagulase genotype (*coa*-type) was determined by multiplex PCR assay as previously described and sequenced for D1 region [26].

### 2.4. Detection of Virulence Factors

For all *S. argenteus* isolates, the presence of 12 staphylococcal enterotoxin (SE) genes (*sea*-*see*, *seg*-*sej*, and *sem*-*seo*), the TSST-1 gene (*tst*-1), exfoliative toxin genes (*eta* and *etb*), leukocidins (*lukDE* and *lukM*), PVL genes, hemolysins (*hla*, *hlb*, *hld* and *hlg*), adhesin genes (*cna*, and *bbp*), and modulators of host defense (*sak*, *chp* and *scn*) were analyzed by multiplex or uniplex PCRs [27,28,29]. Isolates carrying SE genes were further checked to identify the SEs produced using the commercially available Enterotox-F reversed passive latex agglutination (RPLA) kit (Denka Seiken, Tokyo, Japan). This kit incorporates a monovalent antiserum for detecting SEA, SEB, SEC, SED, and SEE. Each culture supernatant was assayed according to the protocol and agglutination was checked after incubation at 25 °C for 18–24 h.

### 2.5. Sequencing of CRISPR/cas Loci of S. argenteus ST2250

Specific primers (Table S4) based on *S. argenteus* type strain MSHR1132 were designed to amplify the CRISPR/cas Loci (clustered regularly interspaced short palindromic repeats (CRISPR)-CRISPR associated proteins (Cas)). CRISPR/cas Loci of selected *S. argenteus* ST2250 isolates were determined by PCR and direct sequencing. Direct repeats (DRs, leader end and inner repeat), degenerated DRs (trailer end repeat), and spacers of the CRISPR region of all 72 *S. argenteus* ST2250 isolates were sequenced to see the polymorphism.

### 2.6. Phylogenetic Analysis

DNA sequences were aligned using the GeneWorks software (IntelliGenetics, Mountain View, CA, USA). The phylogenetic relationships between the species were analyzed using the neighbor-joining method of phylogenetic tree construction, as shown in the MEGA (molecular evolutionary genetic analysis) analytical package [30]. For neighbor-joining analysis, distances between the sequences were calculated using Kimura’s two-parameter model. Levels of similarity were determined between the species. Bootstrap values were obtained for 500 randomly generated trees.

### 2.7. Nucleotide Sequence Accession Numbers

Nucleotide sequences for *dnaJ*, *groEL*, *groESL spacer*, *agrD*, *coa*, and *CRIPSR* have been deposited in GenBank under accession numbers KY995170 to KY995177, and MT542641 to MT542686 (Table S5).

## 3. Results

### 3.1. Identification of S. argenteus by MALDI-TOF MS, MLST Typing and Lack of CrtM

All 96 isolates were recovered from blood cultures and first identified as *S. aureus* by Vitek2 but displayed white colonies (non-pigmented) and lacked the *crtM* gene, pigment production-associated genes, and whole *crt* operon (*crtOPQMN*). All 96 isolates were identified as *S. argenteus* by MALDI-TOF MS using a previously established database [9]. MLST typing identified four sequence types with ST2250 (*n* = 72), ST2793 (*n* = 12), ST1223 (*n* = 10), and ST2198 (*n* = 2) (Table 1). *S. argenteus* ST2250 lineage accounted for 75% (72/96) of isolates. The second most frequent ST was ST2793 (12.5%), and the third one was ST1223.

### 3.2. Antimicrobial Susceptibility and Biochemical Characteristics

All 96 *S. argenteus* isolates were susceptible to methicillin, oxacillin, clindamycin, trimethoprim/sulfamethoxazole, and teicoplanin. Resistance rates for erythromycin (2 isolates), fusidic acid (1 isolate), and gentamicin (2 isolates) were low. Comparison of biochemical activities of the 96 *S. argenteus* isolates tested by VITEK 2 indicated similar results to those of *S. aureus* and a previous study [2], except for urease, *N*-acetyl-d-glucosamine, and d-ribose (Table S1). Of the 96 *S. argenteus* isolates, 62 (65%) were positive for urease, while the overall positivity rate of clinical *S. aureus* (which may include *S. argenteus*) was approximately 2% in the VITEK 2 database and 8.4% in the National Taiwan University Hospital (NTUH) database. For *N*-acetyl-d-glucosamine, 94 (97.5%) isolates were negative, but only 59.8% of *S. aureus* (including *S. argenteus*) were negative based on the results of VITEK 2 in the NTUH. ST1223, which displayed a more than 20% difference from the average positive rate in six biochemical test items, may have exhibited its distinct biochemical profiles more than ST2250 and ST2793 isolates.

### 3.3. Genotyping by Pulsed-Field Gel Electrophoresis (PFGE) and Spa Types

**Figure 1:**
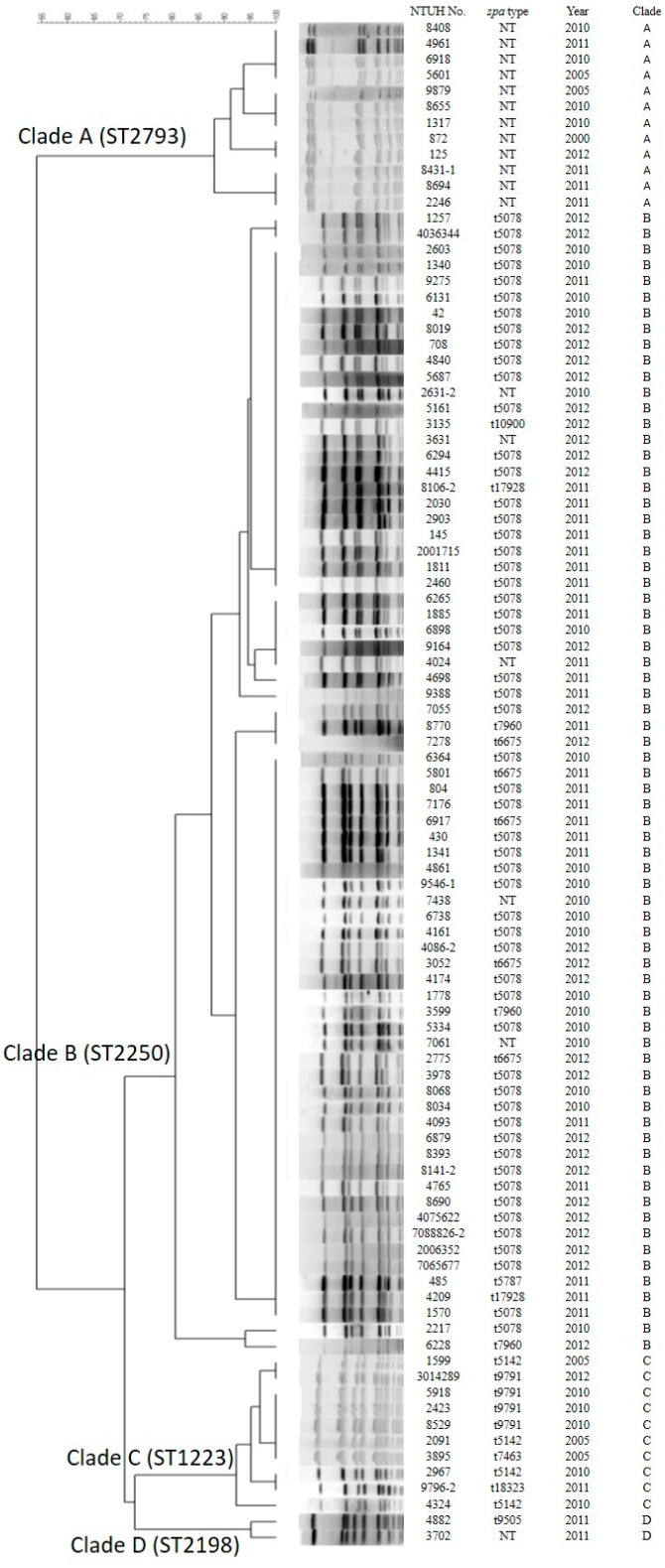
Pulsed-field gel electrophoresis (PFGE) dendrogram of 96 *S. argenteus* clinical isolates. PFGE cluster was assigned to isolates having 80% or greater similarity from the dendrogram. For unknown *spa* type (NT): see Table 2.

PFGE analysis revealed four major pulsotypes (Figure 1). Isolates of the same ST always clustered together. The *spa* types are listed in Table 1 and Table 2. Most *spa* types are t5078 and t6675 among ST2250 and t5142 among ST1223. The other *spa* types have not been reported, and non-typeable (N.T.) (new spa types) in *S. argenteus*. t5078 was the most frequent *spa* type (55 isolates and 57%), followed by t6675 (5 isolates and 5%). However, *spa* was not typeable in 18 isolates, including in all of 12 ST2793 isolates that contain eight isolates with one identical *spa* repeat (t19483).

### 3.4. Phylogenetic Trees of Coa, DnaJ and GroESL and Agr Type

**Figure 2:**
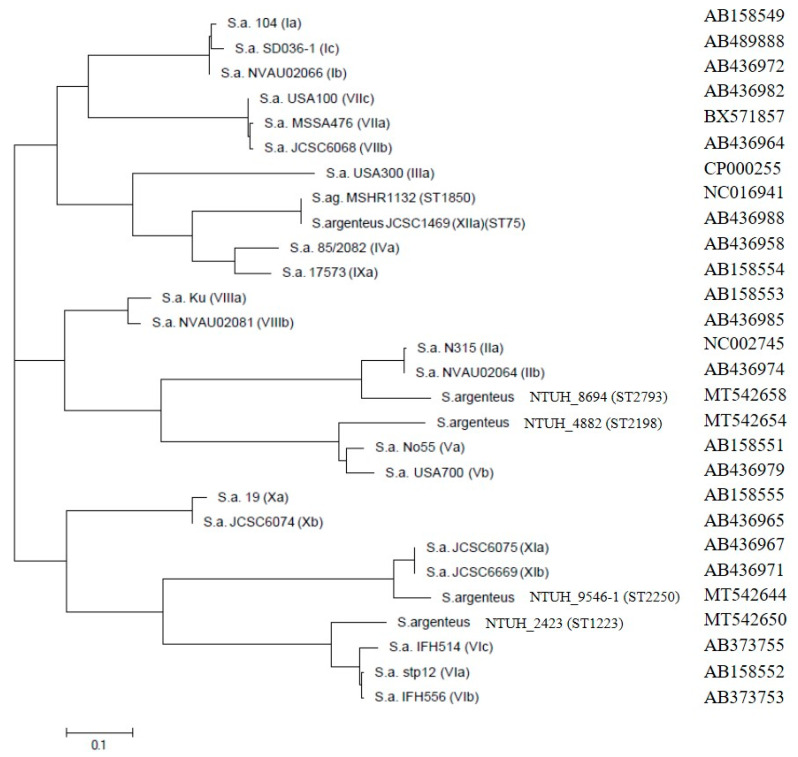

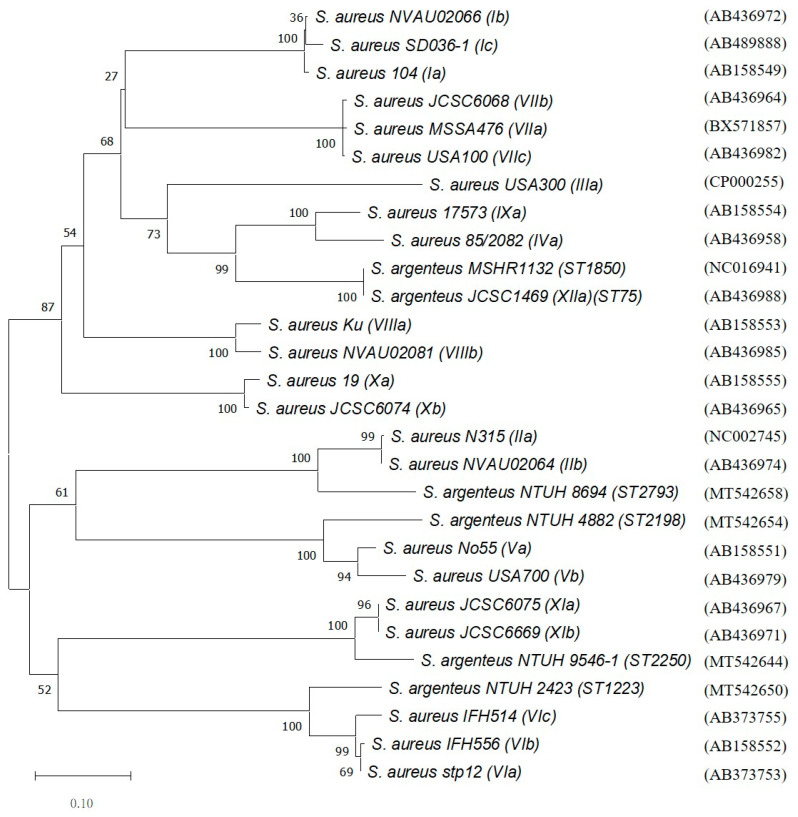
Phylogenetic tree based on D1 region of *coa* gene. The phylogenetic tree was generated using the unrooted neighbor-joining method in the MEGA6 package. The numbers at the nodes are confidence levels, expressed as percentages of occurrence in 1000 bootstrapped resamplings. The scale bar indicates the evolutionary distance between sequences, as determined by measuring the lengths of the horizontal lines connecting two organisms. Each sequence type (ST) type of *S. argenteus* belonged to different clades. The results showed identical partial *coa* sequences at least two isolates for each ST. GenBank accession numbers are given in parentheses with one isolate for each ST in this study.

Selected isolates of each ST of *S. argenteus* were examined for the D1 region sequence of the staphylocoagulase (SC) gene and the (*coa*) gene. Phylogenetic trees showed that each ST type displayed a distinct type (Figure 2). SC genes of *S. argenteus* ST2250, ST2793, ST1223, and ST2198 were close to genotype type XI, type II, type VI, and type V but were assigned to type XId, type XVI, type XV, and type XIV according to the criteria, respectively [31]. In particular, ST1223 has been reported as serotype VI, and the phylogenetic tree of the *coa* gene showed that ST1223 is also close to genotype VI [32].

**Figure 3:**
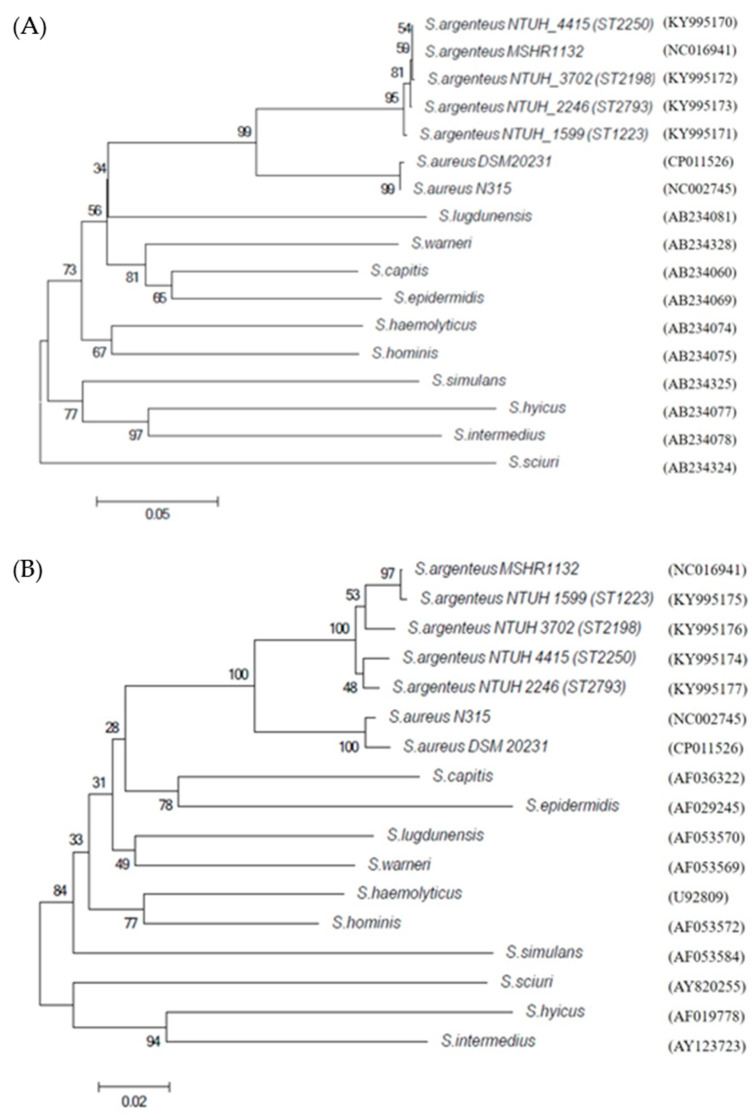
Phylogenetic tree based on partial *dnaJ* (**A**) and *groEL* (**B**). The phylogenetic tree was generated using the unrooted neighbor-joining method in the MEGA6 package. The numbers at the nodes are confidence levels, expressed as percentages of occurrence in 1000 bootstrapped resamplings. The scale bar indicates the evolutionary distance between sequences, as determined by measuring the lengths of the horizontal lines connecting two organisms. The results showed identical partial *dnaJ* or *groEL* sequences at least two isolates for each ST. GenBank accession numbers are given in parentheses with one isolate for each ST in this study.

To further characterize *S. argenteus*, analyses of partial *dnaJ* and *groESL* sequences were performed in selected isolates for each ST. Based on the *dnaJ* and the *groEL* sequences, in this study, the *S. argenteus* clinical isolates and the *S. argenteus* MSHR1132 were clustered together and separated from *S. aureus,* forming a stable clade with bootstrap values of 95% and 100% in the phylogenetic trees, respectively (Figure 3). The BLAST results indicated that *dnaJ* and *groEL* showed the highest similarity (>98%) to that of non-pigmented *S. argenteus* MSHR1132 and approximately 90% similarity to *S. aureus* N315 (Tables S2 and S3). In addition, the spacers between *groES* and *groEL* were analyzed. *S. argenteus* displayed 71 nucleotides of spacer length, while *S. aureus* and *S. argenteus* ST1223 had 75 nucleotides of spacer length (Table 3).

**Figure 4:**
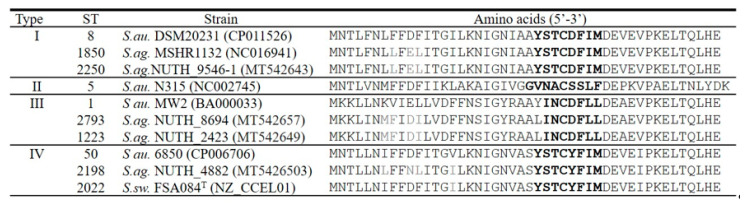
Comparisons of the predicted AgrD amino acid sequences and *agr* types. *S. au.: S. aureus*. *S.ag.*: *S. argenteus*. *S.sw.*: *S. schweitzeri*. AIP (autoinducing peptide) sequences are bolded. The sequences different from *S. au* are grey bottom. The results showed identical partial AgrD sequences at least two isolates for each ST.

Gene *agrD* produces a ribosomal propeptide of which the middle section encodes the seven to nine residue autoinducing peptide (AIP) used as a quorum sensing (QS) signal molecule. The *agrD* alignment is shown in Figure 4. *S. argenteus* harbors *S. aureus arg* types I, III, and IV. The most dominant *agr* are type I (ST1850 and ST2250) and type III (ST1223 and ST2793). *S. argenteus* ST2198 and *S. schweitzeri* ST2022 are known to be of animal origin, and these two isolates belong to type IV. Although *S. argenteus* harbors identical AIP to that of *S. aureus*, *S. argenteus* displays its unique *agrD* amino acid sequences, which are different from those of *S. aureus* or *S. schweitzeri*.

### 3.5. Examination of Virulence Factors

The results of virulence factors are shown in Table 4. Each ST type displayed distinct characteristics. The *hlb*, *hlg*, *luk_PVL_*, *eta*, *etb*, *edin*, *sea*, *sed*, *see*, *seh,* and *tst* genes were not detected in all *S. argenteus* isolates, but the hemolysin genes (*hla* and *hld*) were distributed universally. CRISPR/cas, collagen-binding adhesin gene (*cna*), enterotoxin gene cluster (*egc*), or sialoprotein-binding protein gene (*bbp*) were found exclusively in *S. argenteus* ST2250, ST2793, ST1223, or ST2198 lineage, respectively. Immune evasion cluster (IEC) genes were detected in ~90% isolates, and most of them harbored staphylococcal complement inhibitor gene (*scn*) and staphylokinase (*sak*). In addition, the gene encoding staphylococcal enterotoxin type C (*sec*) was found in 8 out of 12 (67%) ST2793 isolates, and that of staphylococcal enterotoxin type B (*seb*) was found in three S1223 isolates (3/10, 30%) and one ST2198 (1/2, 50%). The expression of *seb* or *sec* in these isolates was further tested by the rapid latex agglutination test. All nine isolates carrying *sec* were exclusively reactive with enterotoxin type C-specific antibodies, but no positive reactions were seen in the isolates carrying *seb*.

### 3.6. Polymorphism of CRISPR/Cas System in ST2250 Isolates

**Figure 5:**
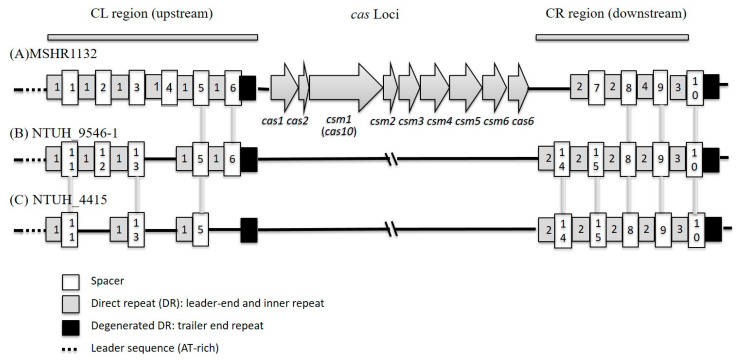
The schematic diagrams of *S. argenteus* CRISPR/cas systems. Sequences of numbers in squares, see Table 4.

Since *S. argenteus* MSHR1132 harbored the CRISPR/cas system, we tried to determine whether *S. argenteus* isolates have the same one. The whole CRISPR/cas system of selected *S. argenteus* ST2250 isolates was determined by PCR and direct sequencing. *S. argenteus* NTUH_9546-1 and NTUH_4415 (ST2250) and MSHR1132 (ST1850) shared the same type III CRISPR/cas system, and the *cas* loci was *cas*1, *cas*2, *csm*1(*cas*10), *csm*2, *csm*3, *csm*4, *csm*5, *csm*6, and *cas*6 in order. (Figure 5) These isolates did not possess the *SCCmec* found in MSHR1132 and displayed different direct repeats (DRs, leader end and inner repeat) and spacers. Upstream (CL region) and downstream (CR region) regions of *cas* loci were sequenced for all 72 ST2250 isolates. A total of four DRs, two degenerated DRs (DDR, trailer end repeat), and 16 different spacers were found in these isolates (Table 5). Half isolates (36 isolates, 50%) displayed the same spacers of CL and CR regions as NTUH_9546-1 isolates (Table 6).

## 4. Discussion

Recent studies have displayed the increasing emergence of *S. argenteus* from many countries, which has been isolated from both humans and animals [33,34,35]. Isolation of *S. argenteus* emphasizes its clinical significance and the importance of correct identification in clinical laboratories [8,15]. MALDI-TOF MS is a simple and accurate method for phenotypic screening and identification of *S. argenteus* [8]. Furthermore, in the present study, the biochemical results of VITEK 2 for the 96 *S. argenteus* isolates showed a higher percentage of urease and were almost negative for *N*-acetyl-d-glucosamine. The results suggest that these two tests also could be screening phenotypes for *S. argenteus*. In the present study, all 96 *S. argenteus* isolates with bacteremia were methicillin-susceptible and comparable to recent reports from Thailand, Japan, and Myanma, while isolates from Europe and Australia were more methicillin-resistant [5,10,14,15,16].

Notably, the earliest and the only *S. argenteus* isolate in the year 2000 was ST2793 with novel *spa* type (t19483). ST2793 lineage is rarely reported in other countries [10,11,12,13,14,36]. To date, ST2793 has been found in Europe and USA but not in Asian counties other than Taiwan [10,13,36]. In addition, a total of 11 new *spa* types across three ST types were first found in the present study. Some known *spa* types were identical to those in Japan and Myanmar isolates [14,16] (Table 2). The t5078 clone occupied more than half of *S. argenteus* ST2250 isolates in Asian countries.

Besides MLST typing and whole genome sequencing, several genetic classification methods have been proposed to distinguish *S. argenteus* from *S. aureus*. PCR-based method targeting the *NRPS* gene, sequencing of the *rpoB* or the *nuc* gene, and lack of the *crtM* gene have been used previously to determine *S. argenteus* [3,7]. The *dnaJ* and the *groEL* genes have been used previously to identify *Staphylococcus* species [23,37]. Phylogenetic analysis based on the *dnaJ* and the *groEL* sequences indicates that these genes may be used as candidate genes for species identification.

Detection of SE-like toxin genes revealed that nine of 12 ST2793 *S. argenteus* isolates carry *sec*. A report from Denmark using whole genome sequencing in four ST2793 and 21 non-ST2793 revealed that none of the isolates carried *sec* [10]. Moreover, the *sec* partial sequence (245 bp) from ST2793 is identical to that of *S. aureus* in the Genbank database and almost identical to sec2 or sec3 genes of *S. argenteus* ST2250 from Japan [14]. ST2250 was described in Thailand as carrying an enterotoxin gene cluster composed of *seC-bov* (enterotoxin C bovine) and *ent*Q (staphylococcal enterotoxin Q), and these two genes should be the two novel superantigens *sel*27 and *se*l26 [14,38]. In addition, some of the ST1223 and the ST2198 isolates in the present study carried enterotoxin type b gene (*seb*) and others did not, which shows the diversity in Taiwan isolates. However, no isolates carrying *seb* were found in Hokkaido, Japan, or Myanmar [14,16]. Interestingly, all ST1223 isolates harbored *egc* clusters as in other studies, but only a small portion carried *seb* gene, which may cause Staphylococcal food poisoning [32,39].

IEC (immune evasion cluster) was carried by bacteriophage and into hemolysin b gene (*hlb*) of Staphylococci [29]. Additionally, 60 isolates of ST2250 carried both *sak* and *scn* (IEC type E), and only one ST2198 isolate harbored *sak*, *scn*, and *chp* (IEC type B), which is comparable to previous studies [11,40]. Regarding other virulence factors analyzed in this study, collagen binding gene (*cna*) and *spa* (protein A) belong to microbial surface components recognizing the adhesive matrix molecules (MSCRAMM) family, and these are associated with bacterial adhesion and pathogenesis [41]. We found that all ST2793 isolates, but not other *S. argenteus* lineages, harbored the *cna* gene and carried new and different *spa* sequences (Table 2). In addition, the ST2793 isolates are methicillin-susceptible, while isolates from Europe carrying *SCCmec* are methicillin-resistant [10]. This may indicate that the ST2793 isolates in Taiwan reveal genetically diverse clones, which is definitely worth further study.

In the present study, all 72 ST2250 isolates harbored the CRISPR/cas system. Previous studies have mentioned the polymorphism of CRISPR/cas system in *S. aureus* and *S. argenteus* [42,43]. However, the present study is the first to study the CRISPR/cas system solely for *S. argenteus* ST2250 lineage, including four direct repeats with 37 bp (DRs, one upstream and three downstream) and 16 different spacers with 33–39 bp (nine upstream and seven downstream) (Table 5). *S. argenteus* ST2250 showed greater diversity of CRISPR/cas than that of most other *S. aureus* ST types and may account for the prevalence and the success of *S. argenteus* ST2250 clones.

In summary, this study focused on the molecular characterization of four ST types of *S. argenteus* isolates in northern Taiwan, finding that *S. argenteus* was isolated as early as the year 2000. Each ST type of *S. argenteus* harbors particular genetic markers that may contribute to their virulence

## Figures and Tables

**Table 1:** *Staphylococcus argenteus* clinical isolates by lineage (*N* = 96).

ST	No. of Isolates Each Year					
*Spa* Type	2000	2005	2010	2011	2012	Total (%)
ST2250	0	0	19	25	28	72 (75.0)
t5078 ^a^	0	0	15	18	22	55 (57.3)
t5787	0	0	0	1	0	1 (1.0)
t6675	0	0	0	2	3	5 (5.2)
t7960	0	0	1	1	1	3 (3.1)
t10900	0	0	0	0	1	1 (1.0)
t17928	0	0	0	2	0	2 (2.1)
Unknown ^b^	0	0	3	1	1	5 (5.3)
ST2793	1	2	4	4	1	12 (12.5)
Unknown ^c^	1	2	4	4	1	12 (12.5)
ST1223	0	3	5	1	1	10 (10.4)
t5142	0	2	2	0	0	4 (4.2)
t7463	0	1	0	0	0	1 (1.0)
t9791	0	0	3	0	1	4 (4.2)
t18323	0	0	0	1	0	1 (1.0)
ST2198	0	0	1	1	0	2 (2.1)
t9505	0	0	0	1	0	1 (1.0)
Unknown ^d^	0	0	1	0	0	1 (1.0)
Total (%)	1 (1.0)	5 (5.2)	29 (30.2)	31 (32.3)	30 (31.3)	96 (100)

^a^ One isolate (NTUH_2217) corresponded to a new single locus variant (*pta* locus 180A > G) of ST2250. ^b,c,d^ See Table 2 for new *spa* type.

**Table 2:** New *spa* types for *Staphylococcus argenteus* clinical isolates.

ST Type	*Spa* Repeat Profile	No. of Isolates
ST2250	299-31-25-17-16-16-16-16	1
ST2250	299-31-25-16-16-16-16-16	1
ST2250	299-31-25-17-119-16-16-16-16	1
ST2250	299-31-17-17-16-16-16-16-16-16-16	1
ST2250	299-31-31-31-25-17-17-16-16-16-16	1
ST2793	259-31-16-16-16-23-17-360-360-25 (t19483)	8
ST2793	259-31-16-16-23-307-360-360-25	1
ST2793	259-31-307-16-23-17-360-360-25	1
ST2793	259-25-25	1
ST2793	259-25	1
ST2198	259-23-23-17-17-17-23-23-23-17-17-16	1

**Table 3:** Nucleotide sequences and length of spacers between *groES* and *groEL* among species. “-” indicated the same nucleotide with MSHR1132 and “.” Indicated blank. The results showed identical spacer sequences at least two isolates for each ST. GenBank accession numbers are given in parentheses with one isolate for each ST in this study.

Strain	Spacer Length (bp)	Spacer Sequence (5′-3′)
*S.ag.* MSHR1132 (ST1850) (NC016941)	71	TACAGAACTTAATTCATAAATAAATTATTAAGAACAATAATCAAACA....TTAAAAAATGGAGGTTTATTATAT
*S.ag.* NTUH_9546-1 (ST2250) (MT542642)	71	-----------------------------------------------....------------------------
*S.ag.* NTUH_8694 (ST2793) (MT542656)	71	--g---g----------------------------------------....------------------------
*S.ag.* NTUH_2423 (ST1223) (MT542648)	75	---------------------------aa------g-a---g-----caat-a--c-------------------
*S.ag.* NTUH_4882 (ST2198) (MT542652)	71	-----------------------------------------------....------------------------
*S.a.* N315 (ST5) (NC002745)	75	--t-a--t-a--------g-------g-a------g-a---g---t-tgac-a--c--------------c--t-
*S.a.* DSM2023*1* (ST8) (CP011526)	75	---------------------------aat-----g-a---g-----caac-a--c--------------c--t-

**Table 4:** Virulence genes of *Staphylococcus argenteus* clinical isolates by lineage ^a^ (*N* = 96).

ST Type (No. of Isolates)	ST2250 (72)	ST2793 (12)	ST1223 (10)	ST2198 (2)
*agr* type ^b^	I	III	III	IV
*coa* genotype ^b^	XId	XVI	XV (serotype VI)	XIV
CRISPR	+ (72)	NT	NT	NT
IEC type ^C^	*sak*+, *scn*+ (60) (Type E) *sak+* (1) *scn+* (1) NT (10)	*scn+* (11) NT(1)	*scn+* (9) NT(1)	*sak*+, *scn*+, *chp*+ (1) *scn+* (1)
*hla* & *hld*	+ (72)	+ (12)	+ (10)	+ (2)
*cna*	NT	+ (12, type III)	NT	NT
*bbp*	NT	NT	NT	+ (2)
*seb*	NT	NT	+ (3)	+ (1)
*sec*	NT	+ (8)	NT	NT
*egc* cluster	NT	NT	+ (10)	NT

^a^ NT, Non-typeable or not detected; The following genes were not detected in any strain: *crtM*, *hlb*, *hlg*, *luk_PVL_*, *eta*, *etb*, *edin*, *sea*, *sed*, *see*, *seh*,and *tst*. ^b^ selected strains (NTUH-9546-1, 8694, 2423 and 3702) for *agr* and *coa* typing, *coa* genotype was based on D1 region and previous described (17) and serotype was according to previous described (27). ^C^ IEC (immune evasion cluster) type B contains *sak*+, *scn*+, and *chp* (60 isolates) and type E contains *sak*+ and *scn*+ (one isolate).

**Table 5:** Direct repeat (DR) and spacer of CRISPR region. DR was classified by core region (underlined, 36 bp).

Type	Sequence	Size(bp)	Note
DR1	GATCGATAACTACCCCGAAGAATAGGGGACGAGAACX	37	upstream
DR2	XATTCGATAACTACCCCGAAGAAGAGGGGACGAGAAC	37	downstream
DR3	TATTCGATAACTACCCCGAAGAAAAGGGGACGAGAAC	37	downstream
DR4	TATTCGATAACTACCCCGAAGAAGAGGAGACGAGAAC	37	downstream
DDR	TGATCGATAACTACCCCGAAGAATAGGGGACAGAGTG	37	Degenerated DR
DDR	TATTCGATAAATACCCCGGAGAACAGGGGGCGAAAAC	37	Degenerated DR
S1	CTACTAAAAAGTTATATGTTTCAACAATTTCGTCA	35	upstream
S2	GGTTTAAGTTTGTCATTATAATCAATCCTTTTTCTT	36	upstream
S3	GATTAAAACGGTTTGCTTTATTTGCATTTAAAATAG	36	upstream
S4	TTTTTCATAGTTAATCAATCCCTTTTCTTTTTT	33	upstream
S5	TAAATCTTTGATTGCTCTTAGCTCTAGTTATGTAT	35	upstream
S6	ACGCTGTAGTGAAGTATAGAAACGGCATGAGTACAAT	37	upstream
S7	TTTTACTGTGTTTTTCATAATTAATCAATCCTTT	34	downstream
S8	TGCCCACTTAATTAATTCATCTAGTCTCATTTCTT	35	downstream
S9	CATCAACTGACTTTTTAACTGTTTTAGTGAATTCGTC	37	downstream
S10	TTAAAGATCTCAACAATAGCGTCCCATATTTTCTG	35	downstream
S11	TAATTGCATTATCAAATGTATATGCTGGATTCCAT	35	upstream
S12	GTACTTAAGATTTCATCAACTTTCTTTTGTACT	33	upstream
S13	CGAATTTTGATTCTTTGTTTGTAAATAATGCTCT	34	upstream
S14	CTATAATAGTTACTGCTTTTGTAACCGTCCATAT	34	downstream
S15	AAATGCTTATCCATTCTAATCATATTTTCAATTTGTTTA	39	downstream
S16	TGCCCACTTAATTAATTCATCTAGTCTTATTTCTT	35	downstream similar to S8

**Table 6:** *Staphylococcus argenteus* Type III-A CRISPR/cas systems.

Isolate ID ^a^ (Accession No.)	Number of Spacers		Direct Repeat
	Upstream	Downstream	
MSHR 1132 (ST1850) (NC016941)	6 (S1~6)	4 (S7~10)	DR1, 2, 3, 4
TD13 (ST2854) (MH513583)	5 (S11~13,5~6)	-	DR1
TD162 (ST2250) (MF167423)	5 (S11~13,5~6)	- ^a^	DR1
SH3 (ST2250) (PRJEB8900)	5 (S11~13,5~6)	5 (S14~15,8~10)	DR1, 2, 3
36 isolates (NTUH_9546-1) (MT542645)	5 (S11~13,5~6)	5 (S14~15,8~10)	DR1, 2, 3
9 isolates (MT542659~60)	4 (S11~13,5)	5 (S14~15,8~10)	DR1, 2, 3
6 isolates (MT542661~62)	4 (S11,13,5~6)	5 (S14~15,8~10)	DR1, 2, 3
5 isolates (MT542663~64)	3 (S13,5~6)	5 (S14~15,8~10)	DR1, 2, 3
3 isolates (MT542665~66)	5 (S11~13,5~6)	4 (S14,8~10)	DR1, 2, 3
2 isolates (MT542667~68)	2 (S11,6)	5 (S14~15,8~10)	DR1, 2, 3
2 isolates (MT542669~70)	4 (S11~12,5~6)	4 (S15,8~10)	DR1, 2, 3
1 isolate (MT542671~72)	5 (S11~13,5~6)	7 (S14~15, 8~9, 8~10)	DR1, 2, 3
1 isolate (MT542673~74)	4 (S11~13,5)	5 (S14~16, 9~10)	DR1, 2, 3
1 isolate (MT542675~76)	4 (S11~13,6)	5 (S14~15,8~10)	DR1, 2, 3
1 isolate (MT542677~78)	3 (S12~13,5)	5 (S14~15,8~10)	DR1, 2, 3
1 isolate (NTUH_4415) (MT542646)	3 (S11,13,5)	5 (S14~15,8~10)	DR1, 2, 3
1 isolate (MT542679~80)	3 (S11,5~6)	5 (S14~15,8~10)	DR1, 2, 3
1 isolate (MT542681~82)	2 (S11,5)	5 (S14~15,8~10)	DR1, 2, 3
1 isolate (MT542683~84)	2 (S5,6)	5 (S14~15,8~10)	DR1, 2, 3
1 isolate (MT542685~86)	4 (S11~13,6)	3 (S8~10)	DR1, 2, 3

^a^ No downstream sequence data of TD13 and TD162.

## Supplementary Materials

Supplementary materials can be found at https://www.mdpi.com/2076-2607/8/8/1157/s1. Table S1: Key biochemical tests used for identification of *S. argenteus* (*N* = 96), Table S2: Partial *dnaJ* (889bp) nucleotide and amino acid sequence similarities, Table S3: Partial *groEL* (780bp) nucleotide and amino acid sequence similarities, Table S4: Primers list for CRIPSR/cas, Table S5: GenBank accession numbers in the present study.
