# Supplementary material for: Heterogeneity of Molecular Characteristics among Staphylococcus argenteus Clinical Isolates (ST2250, ST2793, ST1223, and ST2198) in Northern Taiwan
Source: Microorganisms. 2020 Jul 30;8(8):1157. doi: 10.3390/microorganisms8081157 (PMC7464136; doi:10.3390/microorganisms8081157)
Supplement: Supplementary file 1 [file microorganisms-08-01157-s001.pdf]

Table S1. Key biochemical tests used for identification of S. argenteus (N = 96).

Biochemical Test [a]	ST Type (No. of Positive Isolates)				No. (Positive Rate, %)
	ST2250 (72)	ST2793 (12)	ST1223 (10)	ST2198 (2)	
AMY	1	0	0	1	2 (2.1%)
APPA	0	0	0	0	0 (0%)
LeuA	0	0	0	1	1 (1.0%)
AlaA	0	0	0	0	0 (0%)
DRIB	39	7	3	2	51 (53.1%)
NOVO	1	0	0	0	1 (1.0%)
DRAF	1	0	0	0	1 (1.0%)
OPTO	72	12	10	2	96 (100%)
PIPLC	0	0	0	0	0 (0%)
CDEX	0	0	0	0	0 (0%)
ProA	0	0	0	0	0 (0%)
TyrA	0	0	0	0	0 (0%)
ILATk	71	12	10	2	96 (100%)
NC6.5	72	12	10	2	96 (100%)
O129R	72	11	9	2	95 (99.0%)
dXYL	0	0	0	0	0 (0%)
AspA	0	0	0	0	0 (0%)
BGURr	0	0	0	0	0 (0%)
DSOR	0	0	0	0	0 (0%)
LAC	2	0	0	0	2 (2.1%)
DMAN	72	12	10	2	96 (100%)
SAL	1	2	4	0	7 (7.3%)
ADH1	72	12	10	2	96 (100%)
BGAR	0	0	0	0	0 (0%)
AGAL	4	3	1	0	8 (8.3%)
URE	49	8	3	2	62 (64.6%)
NAG	2	0	0	0	2 (2.1%)
DMNE	72	12	9	2	95 (99.0%)
SAC	72	12	9	2	95 (99.0%)
BGAL	24	0	0	1	25 (26.0%)
AMAN	0	0	0	1	1 (1.0%)
PyrA	71	12	9	2	94 (98.0%)
POLYB	60	11	9	2	82 (85.4%)
DMAL	72	12	10	2	96 (100%)
MBdG	71	12	10	2	95 (99.0%)
dTRE	72	12	10	2	96 (100%)
AGLU	69	5	5	2	81
PHOS	71	12	10	2	95 (99.0%)
BGUR	0	0	0	0	0 (0%)
DGAL	63	10	7	2	82
BACL	71	11	10	2	94
PUL	0	0	0	0	0 (0%)
ADH2S	50	8	4	2	64 (66.7%)

[a] Abbreviation table of Biochemical tests for Vitek 2 GP ID card.

Abbre.	Test	Abbre.	Test
**AMY**	D-amygdalin	**SAL**	Salicin
**APPA**	Ala-phe-pro arylamidase	**ADH1**	Arginine dihydrolase1
**LeuA**	Leucine arylamidase	**BGAR**	Beta galactopyranosidase
**AlaA**	Alanine arylamidase	**AGAL**	Alpha-galactosidase
**DRIB**	D-ribose	**URE**	Urease
**NOVO**	Novobiocin resistance	**NAG**	N-acetyl-D-glucosamine

**DRAF**	D-raffinose	**DMNE**	D-mannose
**OPTO**	Optochin resistance	**SAC**	Saccharose/sucrose
**PIPLC**	Phosphatidylinositol phospholipase C	**BGAL**	Beta-galactosidase
**CDEX**	Cyclodextrin	**AMAN**	Alpha-mannosidase
**ProA**	L-proline arylamidase	**PyrA**	L-pyrrolidonyl-arylamidase
**TyrA**	Tyrosine arylamidase	**POLYB**	Polymixin b resistance
**ILATk**	L-LACTATE alkalinization	**dMAL**	D-maltose
**NC6.5**	Growth in 6.5% NaCl	**MBdG**	Methyl-B-D-glucopyranoside
**O129R**	O/129 resistance (comp.vibrio.)	**dTRE**	D-trehalose
**dXYL**	D-xylose	**AGLU**	Alpha-glucosidase
**AspA**	L-aspartate arylamidase	**PHOS**	Phosphatase
**BGURr**	Beta glucuronidase	**BGUR**	Beta-glucuronidase
**DSOR**	D-sorbitol	**DGAL**	D-galactose
**LAC**	Lactose	**BACL**	Bacitracin resistance
**DMAN**	D-mannitol	**PUL**	Pullulan
		**ADH2S**	Arginine dihydrolase 2

Table S2. Partial dnaJ (889bp) nucleotide and amino acid sequence similarities.

**Strain No.**	**Strain (Accession No.)**	**% *dnaJ* Sequence Similarity [a] (Differences) with Strain：**						
		**1**	**2**	**3**	**4**	**5**	**6**	**7**
**1**	*S.ag.* MSHR1132 (ST1850)(NC016941)	**-**	**99.7(1)**	**100(0)**	**100(0)**	**100(0)**	97.6(7)	97.6(7)
**2**	*S.ag.* NTUH_9546-1 (ST2250) (MT542641)	**99.9(1)**	**-**	**99.7(1)**	**99.7(1)**	**99.7(1)**	97.3(8)	97.3(8)
**3**	*S.ag.* NTUH_8694 (ST2793) (MT542655)	**99.9(1)**	**99.8(2)**	**-**	**99.7(0)**	**100(0)**	97.6(7)	97.6(7)
**4**	*S.ag.* NTUH_2423 (ST1223) (MT542647)	**99.7(3)**	**99.6(4)**	**99.6(4)**	**-**	**100(0)**	97.6(7)	97.6(7)
**5**	*S.ag.* NTUH_4882 (ST2198) (MT542651)	**99.9(1)**	**99.8(2)**	**99.8(2)**	**99.6(4)**	**-**	97.6(7)	97.6(7)
**6**	*S.a.*N315 (ST5) (NC002745)	88.5(102)	88.4(103)	88.6(101)	88.8(100)	88.4(103)	**-**	**100(0)**
**7**	*S.a.*DSM20231 (ST8) (CP011526)	88.4(103)	88.3(104)	88.5(102)	88.6(101)	88.3(104)	**99.9(1)**	**-**

[a]: Data in the upper right portion of the table body indicate nucleotide sequence similarities, and data in the lower left portion of the table body indicate amino acid sequence similarities.

Table S3. Partial groEL (780bp) nucleotide and amino acid sequence similarities.

**Strain No.**	**Strain (Accession No.)**	**% *GroEL* Sequence Similarity [a] (Differences) with Strain :**						
		**1**	**2**	**3**	**4**	**5**	**6**	**7**
**1**	*S.ag.* MSHR1132 (ST1850)(NC016941)	**-**	**100(0)**	**100(0)**	**100(0)**	**100(0)**	99.6(1)	99.6(1)
**2**	*S.ag.* NTUH_9546-1 (ST2250)(MT542642)	**98.1(15)**	**-**	**100(0)**	**100(0)**	**100(0)**	99.6(1)	99.6(1)
**3**	*S.ag.* NTUH_8694 (ST2793)(MT542656)	**98.5(12)**	**98.8(9)**	**-**	**100(0)**	**100(0)**	99.6(1)	99.6(1)
**4**	*S.ag.* NTUH_2423 (ST1223)(MT542648)	**99.7(2)**	**97.8(17)**	**98.5(12)**	**-**	**100(0)**	99.6(1)	99.6(1)
**5**	*S.ag.* NTUH_4882 (ST2198)(MT542652)	**98.7(10)**	**98.1(15)**	**99.0(8)**	**98.7(10)**	**-**	99.6(1)	99.6(1)
**6**	*S.a.*N315 (ST5) (NC002745)	92.8(56)	92.9(55)	93.7(49)	93.6(50)	92.4(59)	**-**	**100(0)**
**7**	*S.a.*DSM20231 (ST8) (CP011526)	92.8(56)	93.0(54)	93.3(52)	92.8(56)	92.4(59)	**98.7(10)**	**-**

[a]: Data in the upper right portion of the table body indicate nucleotide sequence similarities, and data in the lower left portion of the table body indicate amino acid sequence similarities.

Table S4. Primers list for CRIPSR/cas.

Primer Name	Sequence (5′→3′)
cas1 forward_MSHR1132_28-49	CATTACTTTGTTACCGTGAAAG
cas1 Reverse_MSHR1132_28-49	CTTTCACGGTAACAAAGTAATG
cas1 reverse_MSHR1132_880-899	CCCACCTCAATCATTTTAGG
cas1 forward_MSHR1132_880-899	CCTAAAATGATTGAGGTGGG
csm1 reverse_MSHR1132	CAGCCTCCCTTATTTGATAAAC
cas1-R_574-595	CGAATCCATGTAAAGCTAGTTC
Csm2-F	GAAGAGGCAGAGAAAAACAAG
Csm2-R	CCTCCTTTTGATAATATTTAGCG
Cmr4-F	CTGGTTTACACATCGGTG
Cmr4-R	CTCCAACAACTGTCTCG
Csm4-F	GGAAATGACAATAACGTCTG
Csm4-R	CATAGCGATAGACTGGATG
Csm5-F	GGTCCAGTTCATATTGGTAG
Csm5-R	CACATACCTTGCTGGTATG
Csm6-F	GAGATGGTGCAATGCTTC
Csm6-R	GTCTTGTTCATCTATCTCAGG
Cas6-F	CATGGCGTGTTAATGGATTATC
Cas6-R	CCCATGCCTAAGCTTGT
Csm6-R	GTCTTGTTCATCTATCTCAGG
R_MSHR1132_hypothetical protein"71327..71596"	CGGGGTATTTATCGA ATACAG
R_MSHR1132_reverse transcriptase_72125..73651	CGCTATCATTAGCAAATATCC
F_MSHR1132_DEAH box(hypo)_61019..61645	CGAGGATGAAGGAAGAAG
cas1-F_574-595	GAACTAGCTTTACATGGATTCG
csm1-R2	TCGTTGATATTCCTACACC
csm1-endF	CAAACAGATGAACACGGA
csm4-endF	CAACGGCAATGGCTAAAG
cas2_endF	GTGAGAAAAGAACCTAAGCT
Y_csm5-endF	CGATGAAAGTCATTTAGCGA
orfX234F	ACAAGGAAAGATGCTATYTTCC
cas_up_hypo reverse 191-208	CCAAACATCAGGTTCTGC
orfX-break2-F	CTTCAATTTCTGCAGGCG
out-end DEAD-R	ACGTACTGATTCATGGGATG
R_MSHR1132_reverse transcriptase_72125..73651	CGC TAT CAT TAG CAA ATA TCC
down-Cas6-endF	ACAAGCTTAGGCATGGG
down-RT-R	TCTAAAGGACGCCAATCA

Table S5. GenBank accession numbers in the present study.

S. argenteus Clinical Isolates (ST Type)	GenBank Accession No.				
	DnaJ	***GroESL***	***AgrD***	***CoaD1***	***CRISPR***
NTUH_9546-1 (ST2250)	MT542641	MT542642	MT542643	MT542644	MT542645
NTUH_4415 (ST2250)	KY995170	KY995174	-	-	MT542646
14 NTUH isolates (ST2250)	-	-	-	-	MT542659~MT542686-
NTUH_2423 (ST1223)	MT542647	MT542648	MT542649	MT542650	-
NTUH_1599 (ST1223)	KY995171	KY995175	-	-	-
NTUH_4882 (ST2198)	MT542651	MT542652	MT5426503	MT542654	-
NTUH_3702 (ST2198)	KY995172	KY995176	-	-	-
NTUH_8694 (ST2793)	MT542655	MT542656	MT542657	MT542658	-
NTUH_2246 (ST2793)	KY995173	KY995177	-	-	
